# Referee Bias in Professional Football: Favoritism Toward Successful Teams in Potential Penalty Situations

**DOI:** 10.3389/fspor.2020.00019

**Published:** 2020-02-27

**Authors:** Martin Kjeøen Erikstad, Bjørn Tore Johansen

**Affiliations:** ^1^Faculty of Education and Arts, Nord University, Bodø, Norway; ^2^Department of Sport Science and Physical Education, University of Agder, Kristiansand, Norway

**Keywords:** referee bias, social influence, penalty decisions, team success, football

## Abstract

Past studies have indicated that multiple factors may influence sport referees' decisions, such as pressure from spectators and athletes' reputation. Grounded in the social impact theory framework, this study examined whether Norwegian Premier League (NPL) referees are biased by a team's success when awarding penalties. Using video footage (similar to video assistant referees), an expert panel (EP) of four NPL referees evaluated all potential penalty situations (*N* = 43) involving either of two successful teams during an entire NPL season. Fifty-five potential penalty situations from matches without successful teams were also rated. Overall, the match referees identified 73.3% (22 of 30) of the EP-identified penalties during matches without successful teams. Successful teams were awarded 110% (11 of 10) of the EP-identified penalties, while their opponents were awarded 12.5% (1 of 8). Chi square statistic revealed that successful teams were more likely to receive an incorrect penalty compared with their opponents, and less likely to be denied a penalty they should have been awarded. These findings indicate that referees' decisions may be unintentionally biased by a team's success, extending our knowledge about how football referees may be influenced by social forces.

## Introduction

Football (i.e., soccer) referees play an important role in enforcing the rules of the game, evidenced by the ~200 observational and non-observational decisions they make during a single match (Helsen and Bultynck, 2004). Because referees must make decisions under time constraints, in a complex environment and often under ambiguous circumstances, mistakes are guaranteed. Accordingly, referees' decisions may be crucial to a team's potential league win or avoidance of relegation (Baldwin, 2008). Since the referee's fundamental role is to serve as an impartial leader who does not favor one team over another, their mistakes should be equally likely to favor either team. Nevertheless, players, trainers, and spectators all aim to influence referees so they will make mistakes in their team's favor (Di Corrado et al., 2011).

According to a cross-sectional study of Norwegian top-class football referees, most report that their decision-making is unaffected by noise, disturbance, previous mistakes or others' aggressive behaviors (Johansen and Haugen, 2013). Other studies indicate that referees' mistakes are unequally distributed across teams (see Dohmen and Sauermann, 2016 for a review). For instance, Sutter and Kocher (2004) found that referees are more likely to award a penalty to the home team, suggesting that they may be subconsciously influenced by home crowd noise. This is consistent with other findings demonstrating that referees are biased toward the home team when awarding penalties (Nevill et al., 1996; Dohmen, 2008), distributing extra time (Sutter and Kocher, 2004; Garicano et al., 2005; Dohmen, 2008) and administering disciplinary sanctions (Dawson and Dobson, 2007; Buraimo et al., 2010, 2012; Reilly and Witt, 2013). The magnitude of referee bias also appears to be more prominent with increased crowd density (Goumas, 2014), and referees with higher anxiety levels are more prone to the influence of social forces (e.g., crowd noise) compared with less anxious referees (Sors et al., 2019). Thus, despite the intention and perception of impartiality (Johansen and Haugen, 2013), the cumulative evidence suggests that referees' decisions are influenced by social forces.

Aiming to understand referees' decisions, and how they may be biased, Plessner et al. (2009) argued that football referees' decision-making is primarily automatic rather than deliberate, and that it is based on multiple rather than single cues. In addition to direct situational cues, crowd noise is a potential cue that may explain referee home team bias (Unkelbach and Memmert, 2010). Indeed, error management theory (Haselton and Buss, 2000) predicts that decisions made under conditions of uncertainty should favor a bias toward the least costly error (Haselton and Nettle, 2006). For referees, pressure exerted by the home crowd may lead to a home team advantage when decisions are made under uncertainties.

While Mucchi Faina (1996) claimed that the most important form of social influence is conformity to the majority, a second type is influence of abilities and credibility, whereby an individual is influenced by the presence of a skilled and credible person, as determined by factors such as professional success, reputation and knowledge. The effect others have on an individual is explained by Latané (1981) social impact theory, in which others are the sources of impact and the individual is the target, and where this impact is a multiplicative function of the strength, immediacy and number of others. “Strength” refers to the source's power, importance or intensity in relation to the target. Thus, it is reasonable to speculate that a successful team might be an important source of social impact. Consistent with this, focus on potential referee bias in favor of successful teams or athletes has increased. For instance, Findlay and Ste-Marie (2004) found that figure skaters who were known to the judges received higher marks compared with unknown athletes, suggesting a potential reputation bias. However, transferability of these findings to other sports is questionable. Morgulev et al. (2018) found no evidence of a basketball referee bias toward the home team, star players or teams with better reputations, suggesting that referee bias may be context dependent. In the context of football, referee bias has been reported to favor higher level teams; they tend to add more time for higher level teams when they are behind and less time when they are ahead (Lago-Peñas and Gómez-López (2016). Using a statistical approach to analyze whether teams experiencing winning streaks were favored by referees, Audrino (2018) found evidence that one of four teams investigated received preferential treatment from the match referees. However, while a statistical approach is valuable for understanding potential referee bias, it is impossible to evaluate the correctness of referees' decisions without observing those decisions.

In sum, social impact theory (Latané, 1981; Mucchi Faina, 1996) indicates that referees may be targets of social influence by the home crowd (i.e., social pressure from a majority) and/or successful teams (i.e., influences of abilities and status). Yet most referee bias research has focused on a potential home team bias (see Dohmen and Sauermann, 2016). Thus, the purpose of this study was to investigate whether football referees in the Norwegian Premier League (NPL) showed bias based on team success when making important penalty decisions. Based on the evidence described above, we hypothesized that successful NPL teams receive preferential treatment from referees in potential penalty situations.

## Materials and Methods

### Participants

The authors approached the Football Association of Norway, which gave its permission to invite NPL referees to participate in the study. Four male NPL referees were invited by mail and provided written informed consent to participate. These referees were informed that the purpose of the project was to investigate referees' penalty decisions, and that their role was to review potential penalty situations using video footage (like that used by a video assistant referee; VAR). All four participants were active NPL referees, two of whom were also were licensed by FIFA. To ensure their anonymity, no further information about the expert panel (EP) is reported herein.

### Procedures

The study was reviewed and approved by the research ethics committee, Faculty of Health and Sports Sciences, University of Agder. Based on social impact theory (i.e., Latané, 1981; Mucchi Faina, 1996), two NPL teams were identified as potentially impactful based on their status and previous success: Rosenborg and Molde. To identify potential penalty situations, two independent and objective match reports provided by Norwegian National Media were examined for all matches played by Rosenborg or Molde against other NPL teams during a single season (*N* = 56). All situations where one or both reports indicated that the match referee had decided whether to award a penalty were included, regardless of whether the report(s) indicated the accuracy of the decision. The match reports identified 43 potential penalty situations, in which Molde or Rosenborg was the attacking team in 22 matches (14 at home, 8 away) and their opponent was the attacking team in 21 matches (17 at home, 4 away). To identify rates of agreement between the EP and match referees' actual decisions, reports from 118 matches in which other NPL teams had played each other were examined, adding 55 additional potential penalty situations for EP examination; among these, 35 were directed at the home team and 20 at the visiting team. Thus, a total of 98 situations were included.

Video clips of each situation included were gathered and edited using Camtasia Studio software. To reduce potential bias, videos were stopped before it was possible to identify the decision the match referee had made (similar to Plessner and Betsch, 2001). Audio was muted, time and score information was hidden, and the situations were shown from each available angle, zoomed and in slow motion. Finally, the situations were randomized and numbered 1–98. A DVD with these video clips was sent to each EP participant, along with an information letter with instructions to review each situation and mark their judgment on a standardized questionnaire (response options: no foul, free kick to defensive team, penalty). In addition, the EP categorized each situation's difficulty level; response options: easy (1), medium (2) or hard (3). They could pause, rewind and play the situations as needed. As referees of the NPL are trained to objectively evaluate their own performances on video, the EP could evaluate situations in which they had been the match referee. They were also permitted to refrain from evaluating a situation if the video did not show enough information.

### Analysis

Chi square statistic was used to assess discrepancies between individual EP ratings and match referee decisions based on team success status. Situations left blank by the EP were treated as “no foul,” consistent with the procedures used by Nevill et al. (2002).

## Results

The overall agreement between EP and the match referees was 69.4%. Mean EP-rated difficulty was 2.05 (*SD* = 0.50). Among the 55 situations not including a successful team, the EP identified a mean of 30 penalties (range 24–34), while the match referees awarded 22 penalties (13 to the home team and 9 to the visiting team). The overall rate of awarded penalties was 73.3% of the penalties identified by the EP.

Of the 43 potential penalty situations involving the successful teams, the match referee awarded 11 penalties to the successful teams (5 at home and 6 away) and 1 to their opponent (at home). The EP's assessment of the same situations was that that the successful teams should have been awarded 10 penalties (range 8–12) and that their opponents should have been awarded 8 penalties (range 5–11). Over the course of an entire season, the two successful NPL teams were thus awarded 110% (11 of 10) of the penalties identified by the EP, while their opponents were awarded 12.5% (1 of 8). A chi square test revealed that the two successful teams were more likely to benefit from a discrepancy between the EP and match referee, as they received a penalty in 25.5% of the situations the EP would have dismissed compared with the 0% their opponents received [χ^2^ (df = 1) = 15.1, *p* < 0.001]. Furthermore, their opponents were more likely to be denied a penalty that the EP would have awarded, as the match referee dismissed 87.5% of the penalties identified by the EP for successful teams' opponents, compared with 22% for the successful teams [χ^2^ (df = 1) = 30.9, *p* < 0.001]. The EP reported that the situations they judged as penalties to the successful teams were no easier to assess (mean difficulty = 2.2) than those to the successful teams' opponents (mean difficulty = 2.0).

## Discussion

The study objective was to investigate whether NPL referees may be biased by team success when making penalty decisions. The results support the hypothesis that there is a referee bias in favor of successful teams, with the evidence indicating that successful teams are more likely to receive an incorrect penalty compared with their opponents, and that successful teams are less likely to be denied a penalty they should have been awarded.

While consistency between referee- and EP-awarded penalties is ideally 100%, these teams were in fact awarded only 73.3% of the penalties identified by an EP, over the course of a season. Since referees do not always view situations from the best angle, and some situations may have occurred outside the referees' field of view, mistakes certainly occur. Such results are likely explained by the EP having a greater opportunity to identify offenses when viewing the situations on video from different angles, zoomed in and in slow motion. While it is unreasonable to assume that every referee mistake is biased by social forces, the rate of mistakes made by an unbiased referee should be equally distributed among teams (Sutter and Kocher, 2004). However, these data show that successful teams were awarded 110% (11 of 10) of the penalties identified by the EP, while their opponents were only awarded 12.5% (1 of 8).

Error management theory (Haselton and Buss, 2000) predicts that if judgments are made under uncertainty, selection should favor making the least costly error (Haselton and Nettle, 2006). Previous empirical studies have found referee bias in favor of the home team (Sutter and Kocher, 2004; Buraimo et al., 2010), suggesting that referee decisions can be influenced by social forces. Although it was not a primary objective for the current study, the findings did not appear to support previous studies in indicating a referee bias toward home teams. As crowd noise has been highlighted as the main underlying mechanism to explain referee biases toward home teams (see Sutter and Kocher, 2004; Goumas, 2014), such biases are more likely to be valid in the top leagues of Spain (as seen in Garicano et al., 2005; Buraimo et al., 2012), Germany (Sutter and Kocher, 2004; Dohmen, 2008), and England (Nevill et al., 1996; Dawson and Dobson, 2007), where the number of attendances is vastly higher than in Norway. Consistent with recent studies (e.g., Lago-Peñas and Gómez-López, 2016; Audrino, 2018), the present findings indicate that a home team bias is not the only potential referee bias in professional football. Rather, a team's success can be a potentially vital source of social impact on a referee. Indeed, social impact theory (Latané, 1981) explains that impact should be a multiplicative function of the strength, immediacy and number of other people. Although previous studies have tended to focus on a referee bias favoring the home team (e.g., Sutter and Kocher, 2004), the distance from the players to the referee is obviously less, and the players' strength is obviously more powerful than that of the audience. The latter point is particularly relevant to successful teams and players. Accordingly, the players' social influence may have the potential to exceed that of the audience.

These results differ from those reported by Johansen and Haugen (2013), who found that most football referees' decision-making was reportedly unaffected by noise and disturbance, failure in refereeing and aggressive behavior. Although self-reports may be biased, socially desirable responding is more likely to occur when the constructs of interest are sensitive (King and Bruner, 2000); however, the present study should not be used to question whether NPL referees' bias may be intentional. Indeed, we argue that these results are likely explained by referees' decisions being subconsciously influenced by social forces, which has also been proposed by Sutter and Kocher (2004).

The contributions of the study strengths and limitations must be considered. A major strength of this study was that the match referees' decisions were made under actual conditions, where the potential sources of social impact were present; further, these were evaluated by a highly skilled EP, also professional referees, under conditions in which potential social impact was reduced. Since the EP viewed the situation from different angles, zoomed and in slow motion, it is plausible that the panel members had a better opportunity to make a more appropriate decision than did the match referee. It should also be emphasized that the situations used herein included the entire population of potential penalty situations over a season of matches played by two successful teams.

However, the study was not without limitations. Although situations from an entire season were included, the number of potential penalty situations was relatively low, and additional studies will be warranted to allow generalization of these results. Another limitation is the somewhat imprecise categorization of successful teams. While the two teams included herein have been successful over past years and were thus considered simple to identify, a more objective team categorization method is also warranted. Further, a team's success may not fully account for its potential impact on referees, so other factors (e.g., club size) should also be considered. While it is indicated that a player's vocalization can influence the referee to penalize a foul-causing player harder (see Lex et al., 2015), it should also be noted that various players are likely to have different impacts on the referee. Finally, EP decisions should be viewed as indications; their evaluations are also frequently based on ambiguous, unclear situations. Future research could consider investigating potential referee biases in clear (i.e., situations where all EP referees agree) and ambiguous situations (i.e., where EP referees disagrees) separately, rather than to compare actual decisions to individual EP decisions.

## Conclusions

Successful teams' social influence can impact their match referees' important decisions. Given that the results of these decisions may advantage certain teams throughout the season, this issue concerns the fairness of the game overall. Referees' abilities to cope with various sources of social pressure should be considered when evaluating their expertise. Perhaps more importantly, referees' federations should be aware of these findings, and aim to develop relevant training methods so that referees can better resist the influences of social forces. While such programs might include several elements from the sport psychology literature (e.g., mental toughness, self-efficacy, resilience), referees' ability to manage stress and anxiety appear to be particularly relevant, as referees who have high levels of anxiety are more likely to be influenced by social forces compared with referees who have lower anxiety levels (Sors et al., 2019). The findings may also highlight a potential benefit of VAR referees, who should be less exposed to social influences compared with referees on the pitch.

The present study design could be used to examine these effects in other countries in which the sources of social impact are stronger. Experimental studies would also be valuable. For instance, referees might be assigned to either an experimental or control group, to test whether being told that the attacking team is a high-success team makes them more likely to evaluate a situation as a penalty, similar to the study conducted by Jones et al. (2002).

## Data Availability Statement

The datasets generated for this study are available on request to the corresponding author.

## Ethics Statement

The studies involving human participants were reviewed and approved by Research Ethics Committee, Faculty of Health and Sports Sciences, University of Agder. The patients/participants provided their written informed consent to participate in this study.

## Author Contributions

ME developed the study rationale and design, collected and interpreted the data, drafted, and revised the article. BJ contributed to the development of the study rationale and design, interpretation of data, drafted, and revised the article.

### Conflict of Interest

The authors declare that the research was conducted in the absence of any commercial or financial relationships that could be construed as a potential conflict of interest.
